# Personalized Drug Therapy: Innovative Concept Guided With Proteoformics

**DOI:** 10.1016/j.mcpro.2024.100737

**Published:** 2024-02-13

**Authors:** Junwen Su, Lamei Yang, Ziran Sun, Xianquan Zhan

**Affiliations:** Medical Science and Technology Innovation Center, Shandong Provincial Key Medical and Health Laboratory of Ovarian Cancer Multiomics, & Shandong Key Laboratory of Radiation Oncology, Shandong Cancer Hospital and Institute, Shandong First Medical University & Shandong Academy of Medical Sciences, Jinan, Shandong, China

**Keywords:** personalized drug therapy, personalized medicine, proteomics, proteoformics, proteoform, protein drugs, personalized protein drugs, therapeutic protein drug, health risk assessment, cost-effective targeted prevention, artificial intelligence, machine learning, individualized patient profile

## Abstract

Personalized medicine can reduce adverse effects, enhance drug efficacy, and optimize treatment outcomes, which represents the essence of personalized medicine in the pharmacy field. Protein drugs are crucial in the field of personalized drug therapy and are currently the mainstay, which possess higher target specificity and biological activity than small-molecule chemical drugs, making them efficient in regulating disease-related biological processes, and have significant potential in the development of personalized drugs. Currently, protein drugs are designed and developed for specific protein targets based on patient-specific protein data. However, due to the rapid development of two-dimensional gel electrophoresis and mass spectrometry, it is now widely recognized that a canonical protein actually includes multiple proteoforms, and the differences between these proteoforms will result in varying responses to drugs. The variation in the effects of different proteoforms can be significant and the impact can even alter the intended benefit of a drug, potentially making it harmful instead of lifesaving. As a result, we propose that protein drugs should shift from being targeted through the lens of protein (proteomics) to being targeted through the lens of proteoform (proteoformics). This will enable the development of personalized protein drugs that are better equipped to meet patients' specific needs and disease characteristics. With further development in the field of proteoformics, individualized drug therapy, especially personalized protein drugs aimed at proteoforms as a drug target, will improve the understanding of disease mechanisms, discovery of new drug targets and signaling pathways, provide a theoretical basis for the development of new drugs, aid doctors in conducting health risk assessments and making more cost-effective targeted prevention strategies conducted by artificial intelligence/machine learning, promote technological innovation, and provide more convenient treatment tailored to individualized patient profile, which will benefit the affected individuals and society at large.

## Highlights

• Deeply addresses the concepts of personalized medicine and of proteoforms/proteoformics.
• The importance of proteoformics in personalized medicine and betteer drug development.
• Proteoformics benefits scientists to develop drugs and therapies in personalized medicine.

## Graphical Abstract

**Figure:**
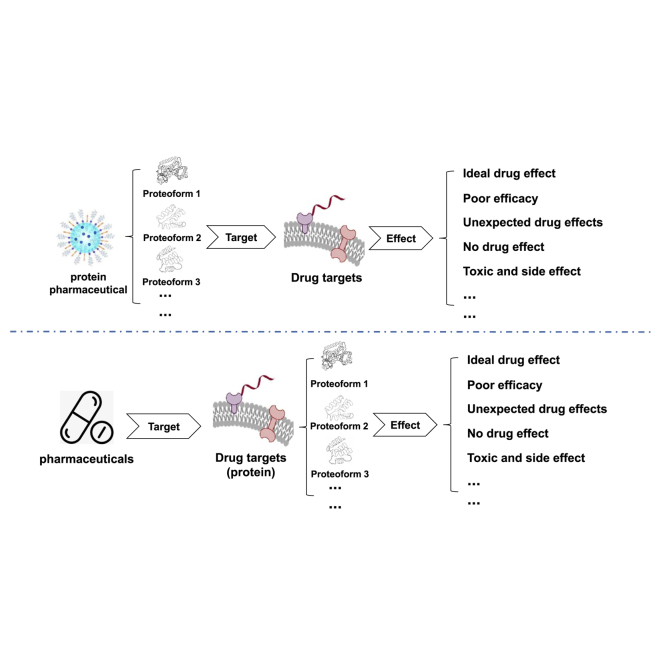

## In Brief

This article in depth describes the concepts of personalized medicine and of proteoforms, why personalized medicine will benefit from proteomics on the level of proteoforms (proteoformics) and explain the importance of proteoformics for development of new and better drugs and diagnostics. It contains many important and helpful aspects of the requirement and application of proteoformics in personalized medicine, which is very important for scientists being involved in the development of drugs and therapies in personalized medicine.

### Personalized Medicine

**Fig. 1:**
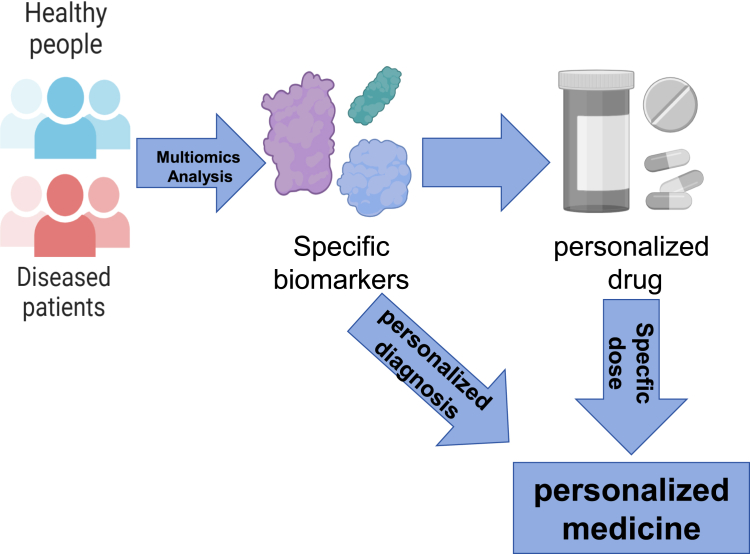
**Diagnostic paths of personalized medicine**.

While it is well-established that patients vary in their response to the same disease (1), a standardized approach is frequently applied in the diagnostic and treatment processes (2). In recent years, the rapid advancement of gene sequencing technology, combined with big data science and bioinformatics, has initiated the development of personalized medicine. As a new medical concept and model that has emerged in the 21st century, personalized medicine has changed the traditional approach (3, 4). The central concept of personalized medicine is to utilize genomics, proteomics, and other omics technologies to examine, identify, verify, and apply biomarkers from large groups of people with specific disease types (Fig. 1). By applying these technologies, it is possible to precisely determine the causes of diseases and their therapeutic targets, along with the accurate classification of distinct disease states and processes. This approach offers healthcare professionals more precise tools to anticipate a patient's susceptibility to a disease, choose the most suitable treatment alternatives, and observe the patient's response to treatment. Personalized medicine aims to offer accurate, individualized treatment to patients and aid in the improved diagnosis, treatment, and prevention of diseases. Through analyzing the genomics, proteomics, and other omics data of each patient, medical professionals can achieve a greater knowledge of how diseases develop, predict patient responses to drugs with greater precision, and formulate personalized treatment plans. Personalized treatment approaches can improve treatment outcomes while reducing the occurrence of unnecessary side effects and drug waste. As technology continues to advance and society provides support, it is anticipated that personalized medicine will expand the horizons of medical care, enhancing patient health and offering more efficient medical treatment (5).

### Personalized Drug Therapy

Various factors contribute to the variation observed in the response to drugs. One commonly cited reason is that the chosen drug treatment may not target the underlying disease mechanism, but is symptomatic treatment because often the disease mechanism could be either unknown or not well understood; and other factors contribute to the variation observed in the response to drugs, including drug interactions, changes in the disease itself (*e.g.*, pathogens are constantly evolving as they replicate in the body (6, 7)), and poor compliance with medication. In addition, it has been observed that treatment nonresponsiveness and adverse drug reactions can differ among individuals of different races or ethnicities, leading to variations in clinical outcomes (8, 9). This highlights the need for personalized drug therapy, which gained significant attention around the year 2000 as a potential solution to this dilemma (10, 11, 12, 13, 14, 15, 16).

Personalized drug therapy is a medical approach that utilizes personal genomic information, such as pharmacogenomics, together with relevant information about the internal environment, such as the proteome and metabolome. By integrating these factors, the goal is to create a tailored treatment plan for each patient that maximizes therapeutic benefits while minimizes side effects. This personalized model has added drug delivery from symptom-based to genotype-based, encompassing all stages of preventing, analyzing, diagnosing and treating disease. The essence of personalized drug therapy is to take into account a patient's genotype, phenotype, and environmental exposures in the development of personalized new therapies and the tailoring of existing medicines. This approach aims to improve drug efficacy, reduce side effects, take the guesswork out of drug use and ultimately achieve the vision of personalized medicine.

### Proteoform and Proteoformics

When it comes to studying proteins, proteoform and proteoformics are two key concepts closely related to protein diversity and related fields of research.

**Fig. 2:**
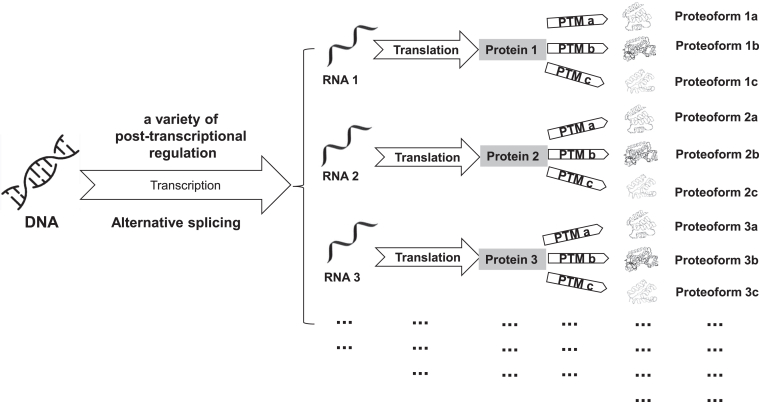
**The reason why the same protein has multiple proteoforms**.

In 2012, the term “proteoform” that refers to the general term for all the different molecular forms of protein products produced by a single gene was first proposed by Smith and Kelleher (17). However, it is worth noting that proteoform has two synonyms “protein species” and “moonlighting proteins”,the terms “protein species” was explained in detail and precisely by Jungblut and Schluter in 2008 and 2009 (18, 19), and “moonlighting proteins” was proposed by Jeffery CJ in 1999 (20). Also, Schluter *et al.* (21) defined the matrix for the comprehensive description of an individual protein species. The morphological diversity of proteins is derived from the influence of factors such as genomic variations, posttranscriptional regulations, and posttranslational modifications (PTMs) (17, 22). For example, mSNPs and other variations in the genome can lead to different forms of proteoforms. In addition, posttranscriptional regulatory mechanisms such as RNA splicing, modification, and proteolytic processing processes, as well as PTMs of proteins, can also lead to protein diversity (Fig. 2). With the development of two-dimensional gel electrophoresis (2DGE) and mass spectrometry (MS), the morphological diversity of proteins is able to better identify and characterize. The complete definition of a proteoform is determined by its binding partners, PTMs, amino acid sequence, spatial conformation, localization, cofactors, and a function (23, 24, 25). The term “proteoform” has been used for 11 years since 2012 (17, 25), leading to the research field of proteoformics that focuses on theories and methods to study the morphological diversity of the proteome (22, 26).

**Fig. 3:**
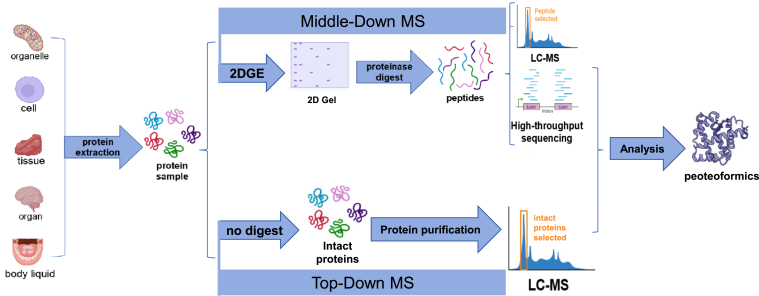
**The research process of proteoformics**.

Proteoformics is a concept first proposed by Xianquan Zhan *et al.* in 2023 (22, 26). The goal of proteoformics is to study the composition and changes of morphological diversity of a canonical protein in the proteome and the impact of this diversity on physiological and pathological processes (22, 26). To achieve this goal, proteoformics utilizes high-throughput analytical techniques and tools to identify, characterize, and quantitatively measure the presence and function of different proteoforms (Fig. 3). More specifically, the research on proteoformics involves the following aspects: First, identification and quantification of proteoforms: the high-throughput methods are used to identify and quantify the existence and relative abundance of different proteoforms. Second, structural analysis of proteoforms: it studies the structure of different proteoforms and their association with function by combining MS and structural biology techniques. Third, functional research of proteoforms: proteoformics research can reveal the functions and interactions of different proteoforms in a cell, tissue, organ, and organism. Finally, it is the data analysis, where bioinformatics and computational tools are being developed to process and interpret large-scale proteoformics data.

The study of proteoformics is crucial for a deep understanding of protein function and disease mechanisms. By revealing protein diversity and regulatory mechanisms, the role of proteins in biological processes can be better understood and provide a more accurate basis for personalized medicine and personalized drug therapy. Therefore, the study of proteoformics will continue to promote our deep understanding of proteoform and lay the foundation for our personalized drug research with the use of proteoforms (but not canonical proteins) as targets.

### Pharmacogenomics

Since the 1950s, it has been suggested that genes control some drug responses. This is based on the association between genetic or racial differences and abnormal drug responses (27). In the decades since then, this concept has been continuously consolidated and strengthened and eventually developed into pharmacogenomics (28, 29, 30, 31, 32, 33, 34).

Pharmacogenomics underpins personalized drug therapy and forms a part of personalized medicine (35, 36). Pharmacogenomics studies how genes impact an individual’s response to specific drugs and can predict adverse reactions and an increased risk of subtherapeutic responses to drugs (37). This relatively new field combines pharmaceutical science represented by pharmacology and gene research and its function based on genomics, with the aim of developing effective and safe therapeutic drugs and their dosages tailored to human individual genetic variation.

Pharmacogenomics describes a method to identify genetic determinants of drug response by analyzing multiple genes or the entire genome. This approach relies on advances in technology and information obtained from the Human genome project, such as high-throughput sequencing, DNA and protein microarrays, and bioinformatics. This field aims to identify the genetic determinants of drug action and eventually utilize genetic testing to improve drug efficacy and minimize toxic side effects. Moreover, comprehending the genetic basis of variable drug response can facilitate the extension of the indications for existing drugs and the development of novel drugs (38).

Pharmacogenomics can facilitate drug discovery and development through two means, including identification of drug targets and development of subpopulation-specific drugs (39). Discovery of drug targets through genomics methods usually initiates the process of designing new drugs. Specifically, this relates to the combination of biomarker-guided molecular targeted therapy drugs and biomarkers. However, as research progresses, it is becoming increasingly clear that genetic biomarkers provide only a partial picture of the key factors that determine the progression of the disease and the response to treatment. In order to achieve the best possible treatment or care, a combination of different types of biomarkers and traditional clinical observations must be used for success. However, significant progress is needed to achieve this goal (40). Subpopulation-specific drug development can also be used to identify genetic polymorphisms that predispose some patients to adverse drug effects, which may occur in only a small proportion of patients treated with a new drug but are toxic enough to prevent further drug development for all patients (39).

### Three Drug Revolutions in History

#### The First Drug Revolution

It took place from the 1930s to the 1960s and was exemplified by penicillin and aspirin, discovered by chance.

Penicillin and aspirin are two significant drugs that have significantly influenced the development of human medicine and pharmacy. The discovery of penicillin can be traced back to 1928, when Alexander Fleming accidentally found a fungal culture that exhibited an antibacterial effect (41). Nevertheless, the discovery and development of the penicillin drug itself was carried out during the 1940s by scientists such as Norman Florey and Ernst Boris Chain (41, 42). Aspirin has a long history dating back to the fifth century B.C. when willow bark was used to alleviate fever and pain (43, 44). The modern form of aspirin was discovered by the German chemist Felix Hoffmann in the late 19th century (37). As a more stable and better-tolerated drug, aspirin was successfully synthesized from salicylic acid. Aspirin is widely used for pain relief and reducing fever and used as an antithrombotic agent (43, 45).

The discovery of penicillin and aspirin had a significant impact on human civilization. Penicillin is the first broad-spectrum antibiotic in the world. Penicillin's discovery allowed humans to fight bacterial infections with more efficiency, reducing the number of deaths caused by infectious diseases. Additionally, it laid the foundation for antibiotic therapy, opening a new era in medicine and fostering advances in this field (43, 44, 46, 47, 48). The discovery of aspirin transformed the approach to pain and fever control, enhancing quality of life, and significantly contributing to the prevention and treatment of cardiovascular illnesses (45, 49). Although neither of these two drugs can be found or invented by people, their development laid the foundation for modern medicine, highlighted the importance of pharmaceutical research, provided important support for our understanding and treatment of diseases, and initiated the first drug revolution.

#### The Second Pharmaceutical Revolution

It occurred from the 1970s until the end of the previous century. Throughout this time, there was a notable progress in medicinal chemistry, which facilitated the development of drugs by pharmacists through compound combination and screening. As a result, tens of thousands of drugs were discovered that significantly impacted human health and well-being.

During this period, some notable drugs were discovered including azidothymidine, an antiretroviral drug used to treat HIV infection and AIDS (50). Prozac is another significant drug, a selective serotonin reuptake inhibitor used to manage psychological disorders like depression and anxiety (51). Beta blockers were also a development aimed at treating conditions like high blood pressure, heart disease, and related cardiovascular ailments (52). Cyclosporine, an immunosuppressant, was discovered to prevent organ transplant rejection (53). Statins were found to effectively lower cholesterol levels, benefiting patients with high cholesterol and cardiovascular disease (54). Insulin analogues were developed to improve blood sugar control in diabetics (55). Angiotensin converting enzyme inhibitors were administered to manage hypertension and coronary heart disease (56). H2 blockers were found to be effective in treating stomach ulcers and acid reflux (57). Selective serotonin reuptake inhibitors for psychological disorders like depression and anxiety (58). Additionally, nonsteroidal antiinflammatory drugs were developed for pain relief and inflammation reduction among other uses (59).

This research development and broad application of compound drugs has greatly benefited humanity. These drugs can treat and prevent diseases, alleviate pain and discomfort, improve quality of life, reduce mortality, and also bring economic and social benefits. These advancements have profoundly impacted human health and well-being, pushing forward the continuous progress of medical science, and leading us into the second pharmaceutical revolution.

#### Modern Genomics–Based Personalized Drug Therapy

It is the way to open the door to the third drug revolution. This novel concept was proposed and introduced by Dr Aaron Ciechanover, the 2004 Nobel Prize winner in Chemistry, during the fifth WLA Laboratories Forum, New Paradigms for Drug Discovery, held on November 4, 2022. The main feature of the third drug revolution is the use of targeted and personalized drugs, which can be designed to target individual's specific genes for more effective treatment. This is of great significance to the medical field to human society as a whole, as it has the potential to revolutionize medication habits and treatment methods of mankind throughout history.

It appears that administering the same treatment to individuals with the same disease can yield varying outcomes, attributed to factors such as age, gender, weight, diet structure, and, more significantly, differences in genetic inheritance that influence people's reactions to the same drug. Sadly, traditional drug development and clinical application tend to be based on the average effect, overlooking the variations between individuals. As a result, personalized drug therapy based on genomics is essential and offers extensive application prospects. The advent of personalized drug therapy heralds the third revolution in medicine and will undoubtedly have a profound impact on traditional medical care while stimulating the development of personalized medicine.

Genomics is at the core of personalized drug therapy. Sequencing and analyzing an individual's genome enable a better understanding of the genetic mutations and variations present, allowing for prediction of their response and tolerance to drugs. For instance, certain gene mutations can reduce the activity of drug-metabolizing enzymes and impact drug metabolism and clearance, leading to excessive or rapid drug accumulation in the body, thereby causing side effects or reduced efficacy. By analyzing the genome of an individual, the correct drug dosage and dosing regimen can be determined to enhance the therapeutic effect and minimize adverse reactions. Moreover, personalized drug therapy can also examine an individual's protein expression and metabolite profile through proteomics and metabolomics analyses to ascertain their physiological status and drug metabolism ability. Such information can aid doctors in making better drug choices, adjusting and optimizing treatment plans based on individual characteristics, and fostering the development of personalized medicine.

In the future, guided by the third drug revolution, the prescription of the appropriate medication will advance toward prescribing the “gene,” or even toward much more precisely prescribing the “proteoform” in future because proteoform is the final structural and functional form of a gene or a canonical protein. Individuals can intervene disease-causing genes to prevent future ailments. Personalized drug therapy development will increase the precision and effectiveness of drug treatments, reduce drug side effects and failure, and enhance patients' quality of life and treatment outcomes. It will propel drug development and clinical practice transformation, extending more personalized and customized medical services for individuals.

## Experimental Procedures

The main experimental procedures of proteoformics in analysis of therapeutic proteins and drug targets in the area of personalized drug therapy include two types of methodologies: top-down mass spectrometry (TD-MS) (60) and 2DGE in combinational with MS (23, 24, 61), which are two complimentary methodologies for proteoformics analysis (Fig. 3).

(i) TD-MS: For this method, the general procedure is that therapeutic proteins or drug-targeted proteins are purified, and the purified proteins are input into mass spectrometer for random fragmentation to form product ions for tandem mass spectrometry analysis. These product ions are used to search database and determine the amino acid sequence of this protein, and their PTMs. TD-MS approach is the commonly used procedure to identify and quantify proteoforms. However, the throughput of this method is not high due to the significant ion inhibitory effect; and currently TD-MS approach is commonly suitable for less than 30 kDa proteins. Currently the maximum proteoform database produced by TD-MS includes about 36,000 proteoforms (62).
(ii) 2DE-MS: It is also called middle-down MS, which includes 2DE-MALDI--MS and 2DE-electrospray ionization (ESI)-MS. The general procedure is that therapeutic proteins or drug-targeted proteins are preferentially separated with 2DE according to their p*I* and relative mass (*M*_*r*_); the 2DE-separated proteins are subjected to enzymatic digestion (such as with trypsin) to form tryptic peptides; then the tryptic peptides are analyzed with MALDI-TOF MS or ESI-ESI-LC/MS. For MALDI-TOF MS analysis, peptide mass fingerprint data are produced and used for searching protein database for protein identification and partial PTM identification. For ESI-LC/MS analysis, tandem mass spectrometry data are produced and used for search protein database for identification of protein amino acid sequence and PTMs. For 2DE-LC/MS approach, it has superhigh throughput in analysis human proteoforms, which can reach up to half million to one million of proteoforms (23, 24, 61, 63).
(iii) 2DE coupled with TD-MS (2DE-TD-MS): For 2DE-TD-MS approach, the common procedure is that the 2DE-separated therapeutic proteins or drug-targeted proteins are purified, and then the purified proteins are subjected to TD-MS analysis. However, currently there is a technical bottleneck for this approach because the 2DE-separated protein is difficult to be separated and purified from the gel. Once this technical bottleneck is resolved, 2DE-TD-MS will be a highly promising, next-generation new proteoformics platform, which will be an innovative breakthrough in the area of proteomics.
(iv) Experimental design and statistical rationale: An appropriate experimental design is necessary for proteoformics study. Proteoformics includes targeted and untargeted approaches.

For targeted proteoformics, for example, when one analyzes the proteoforms corresponding to a given canonical protein or gene, generally one needs to select an appropriate antibody against the canonical protein. This antibody is able to be used to purify the given proteins for TD-MS analysis or this antibody is used to perform 2DE-based Western blot to detect the proteoforms of this given protein or gene and then Western blot–positive proteoforms are used for MS analysis (64, 65). In terms of TD-MS analysis, it is crucial to select a suitable chromatography column. Moreover, stable isotope labeling is also important to quantify each proteoform of a given protein or gene between test and control groups.

For untargeted proteoformics, stable isotope labeling is important to detect and quantify each proteoform between test and control groups. For example, the stable isotope labeling such as tandem mass tag (TMT) is used to label proteoforms in test and control groups, TMT-labeled proteoform samples from test and control groups are equally mixed (24). The mixed TMT-labeled proteoform samples can be used for TD-MS or 2DE-LC/MS analyses to identify and quantify the large-scale proteoforms between test and control groups (24).

Moreover, to guarantee statistical significance of each identified proteoform, at least three reproducible biological samples and/or technological reproducibility are needed for the initial discovery. If proteoform is developed into biomarkers, then much lager sample size will be used, generally several hundred samples will be used. The untargeted proteomics generally produces large-scale proteoformics data, which will need more complex computational biological methodology. Further, if proteoformics data integrate with other omics data, more complex statistics will be needed.

## Contributions of Personalized Drug Therapy

Due to the rising demand for medicine, there has been a substantial change in the range of human diseases. The term “disease spectrum” denotes the occurrence, death rate, and composition of diseases in a particular population and period. This range is not constant but alters as a result of multiple factors like alterations in the environment, lifestyle preferences, populace demographics, and progressions in medical technology. Due to this, the diseases affecting people's lives and health have progressed from mostly infectious diseases to more complex conditions, such as tumors, diabetes, and neuropsychiatric disorders (59, 66, 67, 68, 69). Nevertheless, the development of complex diseases is typically intricate, and there are significant variations in the characteristics of patients, leading to drugs that do not cater to sensitive groups. This, in turn, results in drugs having low average effectiveness and enormous wastage of expenditure. For example, in terms of two patients with lung cancer (70), the same drug may be effective for one patient but not for the other. Furthermore, mutations in the tumor may render the drug gradually ineffective, hence losing its original efficacy. Therefore, personalized drug therapy has extraordinary significance, which is mainly reflected in the following eight aspects:

(i) Improve therapeutic effect: Patients may exhibit varied responses to a single drug. Personalized drug therapy is capable of precisely identifying the most effective drug regimen for patients, based on individual variations in genotype, metabolic capacity, and drug sensitivity. Personalized drug therapy takes into account the patient's genetic variation, metabolic capacity, disease characteristics, and other relevant factors to enhance drug efficacy while minimizing exposure to ineffective drugs.
(ii) Reduce adverse reaction: Patients exhibit varying responses to drugs, with some individuals displaying sensitivity to certain medications, while others experience adverse reactions. Personalized drug therapy has the capacity to anticipate drug susceptibility to adverse reactions based on an individual's genetic information. Furthermore, it can adjust drug dosages or select alternative drugs better suited to individual characteristics to avoid giving patient’s medication that may provoke important adverse reactions, consequently diminishing their likelihood.
(iii) Optimize drug selection: Personalized drug therapy can lead to a better selection of drugs by considering the patient's genotype, protein expression level, metabolic capacity, and other indicators. It can help avoid having to undergo a trial, reduce the chance of treatment failing and increase treatment success.
(iv) Optimize drug dosages: Patients display varying abilities to metabolize and excrete drugs. Personalized drug therapy can customize drug dosage according to factors such as a patient's metabolism, liver and kidney function, among others, to achieve optimal efficacy within the therapeutic concentration range.
(v) Avoid drug interactions: Some patients may be taking more than one medication. Personalized drug therapy can anticipate and prevent possible drug interactions by analyzing the patient's medication list, thus minimizing the chances of adverse events or drug reactions during treatment.
(vi) Accelerate the development of new drugs: Personalized drug therapy offers the potential to more accurately assess the efficacy and safety of new drugs. By analyzing specific patient subgroups and taking individual differences into account in clinical trials, the suitability of new drugs for specific groups can be determined more quickly, thereby accelerating the development and commercialization of new drugs.
(vii) Optimize the utilization efficiency of medical resources: Personalized drug therapy can improve the selection accuracy of drugs, avoiding ineffective treatments and trial and error. It optimizes drug doses and prevents adverse events, thereby reducing medical waste and improving the efficient use of medical resources. This, in turn, reduces overall healthcare costs.
(viii) Promote the development of scientific research: Personalized drug therapy requires the integration of various data, including genomics, genetics, and clinical data, which provide a plethora of resources for precision medicine. Personalized drug therapy has furthered the development of scientific research such as genetic pharmacology, individual genomics, and biomarker discovery, fostering cutting-edge research in the field of personalized medicine.

Thereby, personalized drug therapy is highly significant, particularly in the field of personalized medicine. It enables the creation of more precise, individualized treatment plans for patients, leading to improved therapeutic outcomes, reduced adverse reactions, optimized drug dosages, avoidance of drug interactions, minimized trial and error with drugs, efficient use of medical resources, significant benefits for patients' health and quality of life, and advancement of medical research and innovation. The groundwork for achieving personalized and precise medical goals is thus established. Ultimately, the development and achievement of personalized medicine will be facilitated by personalized drug therapy.

## The Relationship Between Personalized Medicine and Personalized Drug Therapy

Personalized medicine and personalized drug therapy are two approaches that aim to provide patients with more tailored and precise medical services by taking into account their individual differences. These approaches involve a comprehensive understanding of patient’s genetic makeup, physiological conditions, and environmental factors. By analyzing patient’s genes, personalized medicine and personalized drug therapy can identify specific genetic variations that may affect their response to certain medications. This information allows healthcare providers to prescribe drugs that are more likely to be effective and minimize the risk of adverse reactions. Furthermore, these approaches also consider patient’s physiological conditions, such as their metabolism and organ function. By taking into account these factors, healthcare providers can adjust drug dosages and treatment plans to ensure optimal efficacy and safety. Personalized medicine and personalized drug therapy also consider environmental factors, such as lifestyle choices, exposure to toxins, and other external influences, which may influence patient’s health. These factors can include lifestyle choices, exposure to toxins, and other external influences. By understanding these environmental factors, healthcare providers can provide patients with guidance on how to modify their lifestyle or avoid certain environmental triggers to optimize their treatment outcomes.

Personalized drug therapy is a component of personalized medicine, representing its specific implementation in drug treatment. The goal of personalized medicine is to provide tailor-made medical solutions by analyzing various patient data dimensions, such as genome, phenotype, and environmental factors. Selecting the most suitable drug treatment plan based on the patient's genotype, drug metabolism ability, and other relevant information, personalized drug therapy plays a crucial role in this process. Thus, this approach ultimately improves the treatment's effectiveness and safety. Taking into consideration each patient's unique characteristics, personalized medicine optimizes their medical care. Through genetic analysis, medical professionals can identify specific genetic variations that might influence drug response and metabolism. This knowledge enables the selection of drugs that are most likely to be effective and safe for the patient. Moreover, personalized medicine takes into consideration other factors (including lifestyle, environmental exposures, and coexisting medical conditions) to generate a comprehensive treatment plan. Personalized medicine and drug therapy focuses on customized drug selection, dosage, and treatment duration to meet the unique requirements of individual patients, and this forms a crucial element in personalized medicine. This approach factors in the patient's genetic profile, which may influence how drug metabolism operates and interactions occur between drugs and the body. By considering these factors, healthcare providers can optimize drug therapy to maximize its efficacy and minimize any potential adverse effects.

Therefore, personalized drug therapy and personalized medicine are mutually beneficial and interconnected. Personalized medicine cannot exist without personalized drug therapy. Moreover, personalized drug therapy plays a vital role in the field of personalized medicine. Personalized drug therapy contributes to the advancement of personalized medicine by tailoring drug treatments to the specific needs of each patient. Personalized drug therapy and personalized medicine work together to provide patients with more personalized and precise medical services and ultimately enhance treatment outcomes and improve patient’s quality of life.

## Current Typical Personalized Drugs Targeting Protein

**Fig. 4:**
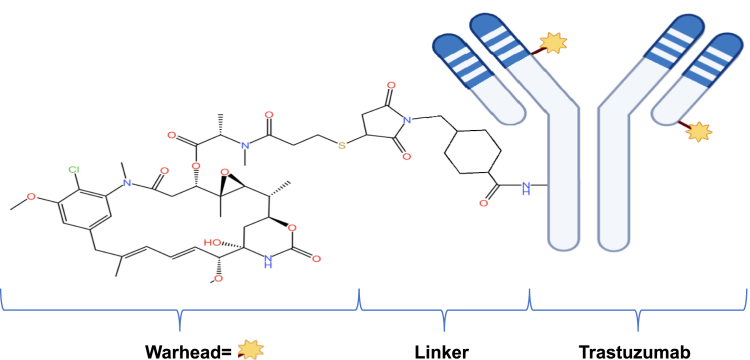
**Molecular formula of Trastuzumab**.

Nowadays, proteomics serves a crucial role in the discovery and design of drugs (71, 72, 73). Proteins are fundamental biological molecules that perform crucial functions in living organisms and contribute to the incidence and progression of multiple diseases (74). Proteomics is the scientific discipline that focuses on analyzing the expression, structure, function, and interactions of proteins across various organisms. Proteomics technology allows for a comprehensive understanding of the structure and function of proteins, which, in turn, illuminates their role in the incidence and progression of diseases (75, 76). In drug development, proteomics can aid in the discovery of new drug targets. By studying disease-related proteins, researchers can determine the key roles played by these proteins in the occurrence and development of diseases thus provides a foundation for drug design. Proteomics has made several critical advances in drug discovery. The following are some types of personalized drugs commonly targeted by proteomics:

(i) Anticancer drugs targeting tumor proteins: To target specific cancer cell surface proteins, it is necessary to identify the key protein targets in tumor cells. This led to the development of targeted proteomics technology. Targeted proteomics is a method that identifies key protein targets associated with tumorigenesis and development, which is performed by analyzing protein expression levels and functions in tumor cells or tissues (77, 78). High-throughput technologies, such as MS, are used to identify and quantify proteins in tumor cells (79). These proteins are analyzed through bioinformatics and functional experiments to determine their functions and relationships. Analysis of the protein expression profile in tumor cells can help to discover key proteins that are closely linked to tumorigenesis and development. These proteins could participate in important biological processes like signaling, cell growth, and the formation of new blood vessels, making them potential targets for therapeutic intervention. An example of such drugs is the drug Trastuzumab (herceptin), which targets HER2 (Fig. 4).

Trastuzumab, also called fam-trastuzumab deruxtecan-nxki in the USA or T-DXd, is an antibody-drug conjugate that targets HER2 specifically (https://www.fda.gov/news-events/press-announcements/fda-approves-new-treatment-option-patients-her2-positive-breast-cancer-who-have-progressed-available). Daiichi Sankyo Company, Ltd and AstraZeneca collaborated in the development of Trastuzumab (http://www.daiichisankyo.com). Trastuzumab (herceptin) is used mainly to treat HER2-expressing cancers, which make up around 20% to 30% of the early-stage breast cancers (https://www.astrazeneca.com/) (80). The discovery of the epidermal growth factor receptor in 1978 and identification of the neu or HER2 gene (also known as ERBB2) in 1984 led to significant research in this area (81, 82). Amplification or overexpression of HER2 was found to be associated with poor survival in patients with breast cancer. This discovery paved the way for the development of trastuzumab, which is a humanized mAb that interferes with the HER2 receptor. The ability of trastuzumab to target HER2 overexpression is considered a major breakthrough in the field of personalized medicine. Trastuzumab offers a more efficient treatment option for patients with HER2-expressing cancers by targeting HER2-positive tumors. It serves as a model for anticancer drugs that target tumor proteins.

(ii) Protease inhibitor (PI): Protease is a specific aspartyl protease encoded by the gene of the human immunodeficiency virus (HIV). Its primary function is to cleave HIV genes and proteins produced by gene expression to form active viral structural proteins and enzymes (83). These proteins and enzymes are essential for the HIV replication process. Before the precursor proteins encoded by retrovirus genes such as HIV can be assembled to complete the virus particles, they need to be cleaved into functional structural proteins under the action of proteases. Proteases play a crucial role in the replication of HIV (84, 85, 86).

PIs are a category of peptide-based compounds that competitively inhibit protease activity or act as inhibitors of complementary protease active sites. These inhibitors primarily act on the last stages of HIV replication. By inhibiting protease activity, they prevent the aggregation and release of DNA formed in the nucleus of infected CD4 cells, and this prevents the cleavage of protein precursors and the formation of mature virus particles. PIs are broadly employed in the treatment of AIDS. The drugs can effectively inhibit viral replication and spread, which can delay disease progression and reduce virus spread. These drugs have achieved clinical success and are now considered important for AIDS treatment. Note that although some trials have reported PI monotherapy as beneficial for maintaining virological suppression in patients, the role of monotherapy has yet to be established (87, 88, 89). Therefore, PIs are usually prescribed as part of antiretroviral therapy, frequently alongside other antiretroviral drugs to enhance effectiveness and minimize the possibility of viral resistance. Moreover, it is necessary to utilize these drugs in strict accordance with the physician's directions to guarantee safety and effectiveness.

(iii) Protein structure modifier: Protein structure modifiers, also known as protein structure-altering compounds, are a group of substances that can directly or indirectly modify the structure of proteins. These compounds can induce conformational changes in the three-dimensional structure of proteins, leading to altered protein function or stability (90). Protein structure modifiers can be natural or synthetic compounds and have different mechanisms of action. Some modifiers can bind to specific regions of proteins, causing conformational changes and modulating their activity (91). The first protein modifier identified was ubiquitin, an abundant and ubiquitous protein that is covalently bound to other proteins either as a marker in proteasomes to guide protein degradation or as a PTM (92, 93). Over the past 20 years, a number of ubiquitin-like protein modifiers have been identified that undergo a similar cascade enzymatic pathway during conjugation, and these modifiers play a critical role in several biological processes and have potential applications in various fields (94). For example, they can be used in drug discovery and development to target specific proteins involved in disease. In summary, protein structure modifiers are valuable drugs that can selectively alter the structure or function of proteins, thereby modulating their activity to achieve the ability to treat disease.
(iv) Immunotherapeutic: Immunotherapy is a medical treatment that utilizes the immune system of the body to combat diseases. It strengthens the human immune system by activating, repairing, or enhancing its function to fight diseases (95, 96). The immune system is the intrinsic defence mechanism of the body which can recognize and neutralize pathogens and tumors. However, under different pathophysiological conditions, the immune system can become imbalanced, leading to a decline in its ability to combat diseases. Therefore, the goal of immunotherapy is to magnify and regulate the immune system so that it can effectively identify and eliminate diseases (97).

The methods of immunotherapy involve the use of immunostimulants, immunosuppressants, and cell therapy. Immunostimulants are commonly used in immunotherapy to activate the immune system and increase its aggressiveness. Protein molecules like interferons and interleukins or immune checkpoint inhibitors such as anti-CTLA-4 antibodies and anti-PD-1 antibodies are used for this purpose (95, 98, 99). The immune system's response can be modulated with immunosuppressants in another type of immunotherapy. This technique is frequently employed in the treatment of autoimmune diseases to alleviate symptoms and regulate the disease progression by suppressing an overactive immune response (100, 101, 102).

Immunotherapy is widely utilized in treating various diseases, particularly in cancer treatment, where it has made significant strides. Immunotherapy allows for targeted treatment of the specific disease, minimum harm to normal cells, and long-lasting healing effects (103, 104). Nevertheless, immunotherapy presents certain challenges and risks. For instance, it can cause undesirable side effects due to excessive immune system activation and some patients may respond differently to the treatment. Therefore, comprehending the unique disease characteristics and individual patient differences and making the appropriate treatment selection and management are essential components of immunotherapy.

The mentioned drugs are some of those that use proteome as drug targets. However, many other drug types are continuously being developed and studied to effectively use proteomic information in creating new drug treatments. Protein-based drug research is a current trend in drug development.

## Disadvantages of the Use of Protein as a Personalized Drug Target

A significant disadvantage of drug targeting in a protein (or proteome) is the possibility of having multiple proteoforms of the same spliced protein. This reflects the fact that a particular protein may undergo variations such as modifications, linkages, or other changes in different cell types, tissues, or disease states, leading to different functions and activities. This diversity gives rise to several challenges for personalized drug focusing on protein (or proteome) as drug targets.

(i) Complexity of proteoforms: Proteomics technologies such as MS and high-throughput sequencing have facilitated the identification and characterization of proteoforms, offering valuable insights into the complexity of the proteome and enabling the separation, identification, and analysis of distinct proteoforms. The complexity of proteoforms refers to the diversity and heterogeneity of a canonical protein that occurs within a biological system. It is acknowledged that distinct proteoforms may have different functions and roles (105). These findings show that a canonical protein is diverse, which limits the accuracy of personalized drugs targeted in a canonical protein (or proteome).
(ii) Target identification and verification: Multiple proteoforms may exist in different cell types or tissues and their expression levels and functions may vary. This suggests that abnormal changes in individual proteoforms could cause the occurrence of some diseases and contribute to individual differences in response to drugs. Therefore, it is necessary to conduct more research and experimental verification of biologically important proteoforms. It is also necessary to verify if a specific proteoform can be used as a personalized drug target instead of a canonical protein.
(iii) Function analysis: The role and regulation of proteins are often associated with their structure and composition. Changes in protein structure and function may occur due to variations in proteoforms, including protein modifications, splicing variants, or mutations (19, 106, 107). Identifying different proteoforms through proteoformics analysis alone is insufficient. Additional experimental studies are necessary to comprehend the functions and regulatory networks of different proteoforms. This may entail expressing and purifying particular proteoforms, followed by functional analyses. Moreover, *via* cellular or animal models, one can explore the role of particular proteoforms in cellular function, signal transduction, and disease. In conclusion, various proteoforms may possess distinct functions and regulatory mechanisms. The comprehension of the functions and regulatory networks of diverse proteoforms necessitates more experimental and functional analyses than mere identification at the proteomic level. In addition, their role in particular diseases should also be probed.
(iv) Drug design and personalized therapy: In the development of personalized medicine or the use of a canonical protein data as drug targets, it is necessary to consider different proteoforms. This is due to potential differences in the structure and function of proteoforms, which can result in diverse responses to drugs. In case of targeting a canonical protein that appears in multiple proteoforms, the drug may be effective on some proteoforms but not on others. This may lead to some modalities having undesirable effects while other modalities may not have the desired therapeutic effect. Such inconsistencies can result in side effects and safety concerns (108, 109, 110). Thus, the development of personalized medicines based on canonical protein (or proteomic) data necessitates a thorough comprehension of the various forms of the target protein and a consideration of their possible variability. This may involve applying precise techniques or research methods to identify and characterize different proteoforms, as well as evaluating their expression levels and functions in particular disease or physiological states. Furthermore, experimental and clinical studies are necessary to ascertain how drugs affect various proteoforms and ensure the safety and efficacy of personalized medicine. In summary, to develop personalized medicine based on canonical protein (or proteomic) data, it is crucial to consider the variability of diverse proteoforms. Drugs with canonical protein targets could have unwarranted or detrimental impacts on various proteoforms, potentially resulting in side effects and safety concerns.
(v) Drug delivery and targeting: First, there may be variances in the intracellular location of distinct proteoforms. Certain proteoforms might localize to various subcellular structures or cell membranes, leading to the requirement for drugs to accurately reach individual proteoforms for effectiveness. Second, there might also be variations in the expression levels and durability of distinct proteoforms. Certain specific proteoforms could express as highly unstable or have low expression levels in cells, constraining the efficient delivery and action of drugs. Variations like these need to be taken into account when one designs drug delivery systems. Moreover, targeting a particular proteoform poses a challenge due to its specificity. Targeting proteoforms can be challenging because of the small differences between them. Specific markers or structural features of proteoforms must be identified to aid drug delivery targeting. Overall, proteoforms may exhibit differences in intracellular location, expression level, and stability, which can pose challenges when targeting them for drug delivery as opposed to canonical proteins.

Generally, research on the use of canonical proteins as drug targets encounters complexity and challenges due to the diversity of proteoforms. As a result, it is crucial to develop personalized medicines that are targeted by proteoformics.

## Current Personalized Drugs Targeting Proteoform

**Fig. 5:**
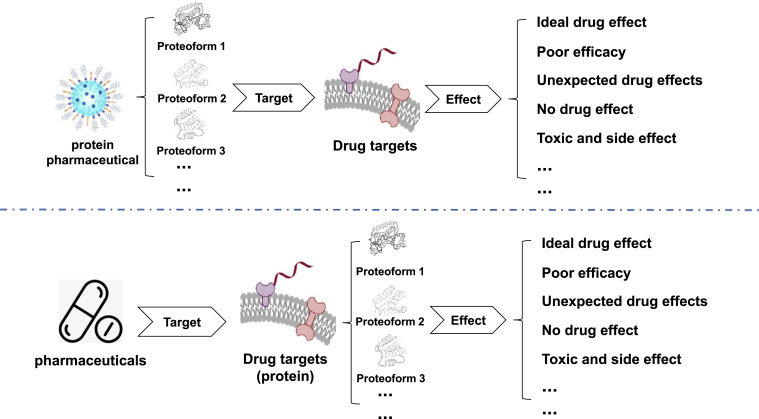
**Effect of proteoform on drug efficacy**.

Although the existence of multiple proteoforms of the same protein was not previously understood, previous examples of differences in proteoform efficacy have been seen during drug development processes. A well-known case is the development of Viagra, which aimed to target the activity of phosphodiesterase (PDE) to enhance cGMP levels, thereby decreasing intracellular calcium ion concentration and relaxing the vasculature. Smooth muscles and lowering blood pressure make it a new antihypertensive and angina drug. Interestingly, PDEs exist in multiple proteoforms, and the PDE targeted by Viagra is actually cGMP-specific PDE type 5, which is widely distributed in the corpus cavernosum, not the nonspecific cGMP found in vascular smooth muscle as expected. Viagra lacks the anticipated antihypertensive and angina effects but has achieved recognition as a wonder drug for managing erectile dysfunction. Due to the impact of different proteoforms on drug efficacy, a drug has other unexpected pharmacological effects, just like Viagra has a pharmacological effect in treating erectile dysfunction, which is undoubtedly acceptable. However, ones should also think deeply that if a drug not only fails to produce the desired efficacy due to differences in proteoforms, but worsens the patient's condition or causes other toxic and side effects, thereby creating potential safety issues, this is undoubtedly what we do not want to see and want to avoid (Fig. 5). This is a wake-up call for ones to fully realize that when designing drugs, ones should pay full attention to whether the drugs ones develop and the drug targets they act on have multiple proteoforms and the potential impact that differences of these proteoforms may have on drug efficacy.

When designing pharmaceuticals, receptors *in vivo* are a key factor in our search for drug targets. Amongst these receptors, G protein–coupled receptors are currently a focus of research. However, the majority of G protein–coupled receptors exist in various proteoforms. For instance, the receptors of the efferent nervous system are presently a topic of intensive study. The efferent nervous system's receptors respond to transmitters based on their selectivity, which is divided into cholinoceptor and adrenoceptor. These two types are further classified into M receptors, N receptors, and alpha receptors, beta receptors. Due to genetic coding differences, all of these receptors have various proteoforms. Previous studies have established that distinct proteoforms are distributed across different areas of the human body and result in varying effects (Table 1). This is not a chance occurrence but rather a widespread characteristic of several drug targets located in the human body. Despite being identical proteins, they exhibit distinct proteoforms due to differences in genetic encoding and other factors, leading to dissimilar effects. When designing drugs and selecting the intended target protein, it is essential to consider the impact of the current proteoform. Ones should, or indeed must, target the drugs ones we design to the specific proteoform, rather than just targeting a generic protein. This will help avoid changes in drug efficacy caused by differences in different proteoforms.

**Table 1:** Different proteoforms of receptors from efferent nervous system and their different effects

Effector	Receptor of efferent nervous system					
	Adrenoceptor			Cholinoceptor		
	Proteoform	Effect	Ref.	Proteoform	Effect	Ref.
Eye						
Pupil opening muscles	α_1_	Contract (delate pupils)	(118)			
Pupil sphincter				M_**3**_	Contract (miosis)	(119)
Ciliary muscle	β_2_	Slack (farsightedness)	(118)	M3	Contract (shortsighted)	(120)
Heart						
Sinus node	β_1_	Increased autorhythmicity and accelerated heart rate	(121)	M2	Decreased autorhythmicity and slowed heart rate	(122)
Room knot	β_1_	Speed up conduction	(123)	M_**2**_	Slow down conduction	(124)
Conduction system	β_1_	Speed up conduction	(125)	M_**2**_	Slow down conduction	(126)
Myocardium	β_1_	Increased contraction	(127)	M_**2**_	Contraction weakened	(128)
Vascular smooth muscle						
Skin, mucosa	α_1_, α_2_	Contract	(129)			
Abdominal viscera	α_1_, β_2_	Contract, relax	(130)			
Coronary blood vessels	α_1_, β_2_	Contract, relax	(131)			
Skeletal muscle	α_1_, β_2_	Contract, relax	(132)	M_**3**_	Relax	(133)
Brain	α_1_	Contract	(134)			
Kidney	α_1_, β_2_	Contract, relax	(135)			
Lung	α_1_, β_2_	Contract, relax	(136)			
Endothelium				M_**3**_	Releases NO	(137)
Bronchial smooth muscle	β_2_	Relax	(138)	M_**3**_	Contract	(139)
Stomach and intestines						
Smooth muscle	α_2_, β_2_	Slack	(140)	M_**3**_	Contract	(141)
Sphincter	α_1_	Contract	(142)	M_**3**_	Slack	(143)
Gallbladder and biliary smooth muscle	β_2_	Relax	(144)	M	Contract	(145)
Bladder						
Uroid muscle	β_2_	Slack	(146)	M_**3**_	Contract	(147)
Sphincter	α_1_	Contract	(148)	M_**3**_	Slack	(141)
Uterine smooth muscle	α_1_，β_2_	Contract, slack	(149)	M_**3**_	Contract	(150)
Salivary gland	α_1_	Secrete K^**+**^ and water	(151)	M_**3**_	Secrete K^+^ and water	(152)
	β	Secret salivary amylase	(151)			
Bronchial gland	α_1_，β_2_	Decreased secretion, increased secretion	(153)	M_**3**_	Increased secretion	(154)
Acid gland (wall cells)				M_**1**_	Increased secretion	(155)
Skin sweat glands	α_1_	Local secretion (palms of hands and feet)	(156)	M_**3**_	Secrete	(157)
Paranephrocyte	β_1_	Secrete renin	(158)			
Adrenal medullary				N_**M**_	Secrete	(159)
Metabolism						
Hepatic gluconeogenesis	β_2_, α_1_	Increase	(160)			
Hepatic glycogen decomposition	β_2_, α_1_	Increase	(161)			
Fat decomposition	β_3_	Increase	(162)			
Skeletal muscle	β_2_	Break down glycogen	(163)	N_**M**_	Contract	(164)

## New Development of Personalized Drug Under Proteoformics

**Fig. 6:**
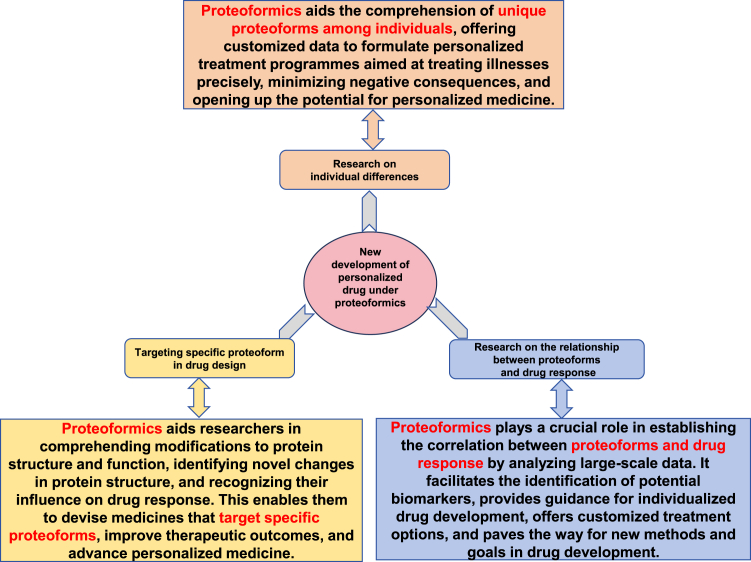
**New development of personalized drug under proteoformics**.

Proteoformics is to study different proteoforms derived from a canonical protein, which has significant implications for the development of personalized drugs. Proteins form the basis of numerous functional molecules in living organisms, and variations in their structure and function are essential factors in the development of diseases and response to drugs. Drug design has typically relied on the structure and function of particular proteins, which are considered to be units with stable structures and functions. Recent studies have demonstrated that there may be numerous variations for each canonical protein. Several factors, including protein modifications, protein isoforms, and protein appendages, can contribute to these variations. Hence, when dealing with proteins that possess multiple proteoforms, ones must take into account the structural and functional discrepancies among different proteoforms derived from a canonical protein. This ensures that the medications are effective against specific proteoforms. Recent progressions in proteoformics enable researchers to comprehend more comprehensively each proteoform of a canonical protein and contribute toward tailored drug development. In particular, the new progress in proteoformics for tailored drug development encompasses the following essential aspects (Fig. 6):

(i) Research on individual differences: Proteoformics may assist researchers in comprehending differences in proteoforms of a canonical protein. Proteoformics technology allows researchers to detect PTMs of diverse proteins, including phosphorylation, acetylation, methylation, ubiquitination, nitration, nitrosylation, and others. With this personalized data, physicians can create customized treatment plans tailored to the patient's proteoformic profile. As a result, drug therapy can be more precisely focused on the patient's disease mechanism, which improves the efficacy of therapy and decreases side effects. Taken together, proteoformics provides the potential for creating personalized medicine based on researcher’s comprehension of proteoform discrepancies between individuals. Anticipated outcomes include more precise and effective approaches for predicting, diagnosing, and treating diseases.
(ii) Targeting specific proteoforms in drug design: The application of proteoformics can guide researchers in comprehending protein structure and functional changes. The utilization of proteoformics enables researchers to recognize and examine various protein structure alterations, including protein isoforms, mutants, or variants with modifications. Such structural variations can significantly impact the protein's functionality. Comprehending the effect of specific protein structure variations on drug response can facilitate designing medications that aim to target precise proteoforms. For instance, particular modifications of proteins can augment or diminish the connection between these proteins and a drug. By utilizing this comprehension, scientists can formulate drugs that specifically aim at particular protein modifications, producing a rise in drug effectiveness and decreasing possible negative effects. Thereby, proteoformics can assist researchers in gaining a more detailed comprehension of the structural and functional differences within proteins. This information can then be used to design drugs that aim at specific proteoforms. Such applications can enhance the precision and efficiency of drugs and accelerate the progression of personalized medicine.
(iii) Research on the relationship between proteoform and drug response: Proteoformics can assist researchers in establishing the relationship between proteoform and drug response, offering guidance for developing personalized drugs. By analyzing large-scale proteoformics data, researchers can determine and quantify the correlation between particular proteoforms of a protein and drug response. These proteoforms of a protein may take the form of genetic variations, posttranscriptional modifications, or other alterations in protein structure and function. Researchers can link data on drug response (such as drug efficacy, adverse reactions, etc.) with proteoformics data to identify proteoforms that are related to drug response. This process helps to identify potential biomarkers, which are indicators of distinct patterns of proteoforms that predict drug response. The results of these association analyses could offer essential guidance toward the development of personalized medicine. Based on how specific proteoforms relate to drug response, researchers could design customized treatments for different patient populations. This could involve drug dose optimization, appropriate drug combination selection, and even customizing specific drugs to intervene with particular proteoforms. Furthermore, by establishing the correlation between proteoformic data and drug response, researchers could offer new objectives and methodologies for drug discovery and development. Proteoformic data can uncover new protein variations or modifications linked to drug response, hence inspiring novel drug development targets. Proteoformics can assist researchers in establishing the correlation between proteoformics and drug response, offering crucial guidance for individualized drug development and enhancing innovation in drug discovery and development.

Proteoformics generally offers comprehensive and detailed protein information to aid in the development of personalized medicine, enhance researcher’s comprehension of protein function and variation, and provide more accurate guidance to develop personalized medication. As a result, individualized drug treatments will be promoted, and the effectiveness and safety of drugs will be improved.

## Conclusion

Traditional drug development and treatment methods typically design and promote drugs based on group average effects, thereby ignoring individual differences. Nonetheless, personal characteristics of each individual, such as genes, physiological conditions, environment, and lifestyle, can significantly impact drug responses. As a result, the idea of personalized medicine has gained widespread interest in the medical field. Personalized drug therapy, as a crucial component of personalized medicine in the pharmaceutical area, is instrumental in advancing the development of personalized medicine.

To understand the relationship between specific genotypes and drug metabolism, mechanism of action, or efficacy, personalized drug therapy necessitates research in genomics, genetics, and biomarkers. By analyzing and interpreting individual genomes, doctors and pharmacists can predict the patient's drug response, adjust dosage, choose patient-tailored drugs, and optimize the treatment plan to achieve the aim of personalized medicine with increased accuracy. The benefit of personalized drug therapy is increased treatment accuracy and effectiveness and reduced adverse reactions and side effects. Personalized drug therapy can assist physicians in the proper selection of drugs and the adjustment of dosages and treatment schedules to match individual needs and personal characteristics. Personalized drug therapy can also aid in the management of multidrug interactions and reduce negative interactions among drugs. However, personalized drugs, particularly those focusing on canonical proteins, often only examine the amino acid sequence decoded by the protein's genome, disregarding its various forms. This approach results in certain problems and drawbacks, as the same protein might contain diverse proteoforms arising from genomic variation, posttranscriptional modifications, and PTMs, and these variations may be closely linked to the onset and progression of the disease. Additionally, distinct proteoforms vary significantly regarding their structure and function, making it challenging to design, deliver or target personalized drugs focused on canonical proteins (or proteome). Protein’s dissimilar proteoforms may have different interactions and functions, promoting the need to target these particular proteoforms for the development of personalized drugs suited to personalized drug therapy, which can provide a more exact and focused treatment strategy for personalized medicine.

Overall, personalized drug therapy is a crucial element of personalized medicine and a leading clinical treatment method for the future. It offers ones more precise, efficient, and safe options for treatment. The improved proteoformics research enables better study of individual differences and clearer understanding of the relationship between proteoforms and diseases. This supports the design and development of drugs for specific proteoforms and improves understanding of individual differences in drug response, enabling the creation of tailor-made treatment plans for each patient. Such an approach enhances the treatment effects, reduces adverse reactions, accelerates new drug development and scientific research, and provides vital supports for the practice and application of individualized medicine.

## Outlook and Expert Recommendations

Personalized medicine and drug therapy have made significant advancements. Integrating advanced technology with traditional experience can reduce damage and retain precision. Advancements in biotechnology, including human genetics and genomics, gene sequencing technology, signal pathway research, gene interaction and network, and molecular regulation, will drive the future development of personalized medicine. Personalized drug therapy can make personalized medicine the dominant medical model in the future, and the personalized drug research and development model might become the international standard and high-end point of biomedical technology.

For the development of personalized medicine, we strongly recommend to study personalized medicine targeting proteoforms as the drug target. Due to the advancements in 2DGE2DE-MS and TD-MS, we are aware of the effect of proteoforms on individual clinical differences. Moreover, we believe that personalized medicine targeting proteoforms is essential for predictive, preventive and personalized medicine (PPPM) and precision medicine because of three significant implications: First, in-depth study of proteoform's specificity can improve understanding of disease mechanisms, which can reveal pathophysiological processes, discover new drug targets and signaling pathways, and provide a theoretical basis for developing new drugs (111, 112). Second, the detection of individual patient’s proteoform levels can devise targeted treatment strategies. In terms of differences of proteoforms for different individuals, individualized selection of drug targets and treatment methods can improve therapeutic effects, reduce side effects, and help physicians to conduct health risk assessment and make more cost-effective targeted prevention (113, 114). In addition, proteoformics can promote 3P medicine to better conduct artificial intelligence/machine learning to promote technological innovation and more convenient treatment for personalized patients (115, 116, 117). Proteoforms as a drug target can potentially stimulate innovation in drug development. The conventional drug development approach primarily concentrates on the amino acid sequence of protein target; however, neglects the importance of protein morphology and modifications. The advancement of personalized medicine can bolster the research and development of proteoformics, which will contribute to the discovery of a new generation of targeted drugs. In general, the use of proteoform as a drug target in personalized medicine creates new opportunities and difficulties for the development of PPPM and precision medicine. This approach can reinforce the research and comprehension of canonical proteins, enhance the precision and effectiveness of drug treatment, stimulate innovation in drug development, and facilitate more options for individualized treatment. We propose proteoform-targeted personalized drugs to be developed based on the following three areas:

(i) Target-specific protein modifications in drug design: Protein modification is a vital pathway for protein function and regulation. The pattern of protein PTMs can differ between individuals and in various disease states. Proteoformics help identify protein modifications linked to specific diseases, which makes it possible to develop drugs that specifically target these modifications. These drugs regulate protein PTM levels and consequently influence the functions of related signaling pathways or metabolic pathways.
(ii) Target protein isoforms in drug design: Protein isoforms are different forms of proteins produced by a single gene exhibiting structural and functional variations. Proteoformics assists to identify protein isoforms found in distinct individuals and comprehend their contributions to the onset of diseases. Researchers can design drugs targeting a certain protein isoforms to attain individualized therapeutic benefits.
(iii) Investigate the association between proteoforms and drug response: Understanding the connection between proteoformics data and drug response can assist to create customized medications depending on protein variations. Large-scale proteoformics data enables to identify proteoform signatures that associate with drug response to develop individualized treatment. Individualized medicine can be adapted based on individual’s proteoformics profile to improve efficacy of treatment and reduce harm.

In summary, personalized drug therapy offers significant support for advancing personalized medicine. Development of bespoke drugs with the use of proteoforms as drug targets can refine treatment for PPPM and precession medicine. Protein modifications, isoforms, and individual proteoformics associated with drug responses can enable custom drug design, improve drug treatment outcomes, and provide more precise treatment approaches for individuals. This type of personalized medicine is anticipated to usher in a new epoch of PPPM and precision medicine and stimulate the development of more refined, effective, and fewer side effect individualized treatments to cure diverse ailments.

## Data Availability

All data and materials are provided in this article, which can be available publicly.

## Conflict of interest

The authors declare no competing interests.

## References

[1] Dewey F.E., Gusarova V., O'Dushlaine C., Gottesman O., Trejos J., Hunt C. (2016). Inactivating variants in ANGPTL4 and risk of coronary artery disease. N. Engl. J. Med..

[2] Abul-Husn N.S., Kenny E.E. (2019). Personalized medicine and the power of electronic health records. Cell.

[3] Ginsburg G.S., McCarthy J.J. (2001). Personalized medicine: revolutionizing drug discovery and patient care. Trends Biotechnol..

[4] Jain K.K. (2002). Personalized medicine. Curr. Opin. Mol. Ther..

[5] Washington D.C., The National Academies Press (2011).

[6] Thannickal S.A., Spector S.N., Stapleford K.A. (2023). The La Crosse virus class II fusion glycoprotein ij loop contributes to infectivity and replication *in vitro* and *in vivo*. J. Virol..

[7] Zhang B., Xu S., Liu M., Wei Y., Wang Q., Shen W. (2023). The nucleoprotein of influenza A virus inhibits the innate immune response by inducing mitophagy. Autophagy.

[8] Mega J.L., Close S.L., Wiviott S.D., Shen L., Hockett R.D., Brandt J.T. (2009). Cytochrome p-450 polymorphisms and response to clopidogrel. N. Engl. J. Med..

[9] Panattoni L., Brown P.M., Te Ao B., Webster M., Gladding P. (2012). The cost effectiveness of genetic testing for CYP2C19 variants to guide thienopyridine treatment in patients with acute coronary syndromes: a New Zealand evaluation. Pharmacoeconomics.

[10] Jelliffe R. (2000). Goal-oriented, model-based drug regimens: setting individualized goals for each patient. Ther. Drug Monit..

[11] Daly A.K. (2007). Individualized drug therapy. Curr. Opin. Drug Discov. Dev..

[12] Spigset O. (2000). Fra konfeksjon til skreddersøm--fremtidige muligheter for individuelt tilpasset legemiddelbehandling from ready-made to tailor-made--future possibilities for individualized drug therapy. Tidsskr. Nor. Laegeforen..

[13] Aarbakke J. (2000). Who will receive future tailor-made drugs?. Tidsskr. Nor. Laegeforen..

[14] Steen V.M. (2000). Skreddersøm av legemidler i et nytt millennium tailor-made drugs in the new millennium. Tidsskr. Nor. Laegeforen..

[15] Nohmi T. (2002). Drug discovery based on genomic information. Rinsho Byori.

[16] Ferriols Lisart F., Ferriols Lisart R. (2003). Pharmacogenetics: where are we and where are we going to?. Farm. Hosp..

[17] Smith L.M., Kelleher N.L. (2013). Consortium for top down proteomics. Proteoform: a single term describing protein complexity. Nat. Methods.

[18] Jungblut P.R., Holzhütter H.G., Apweiler R., Schlüter H. (2008). The speciation of the proteome. Chem. Cent. J..

[19] Schlüter H., Apweiler R., Holzhütter H.G., Jungblut P.R. (2009). Finding one's way in proteomics: a protein species nomenclature. Chem. Cent. J..

[20] Jeffery C.J. (1999). Moonlighting proteins. Trends Biochem. Sci..

[21] Schluter H., Riedner M., Jungblut P.R. (2008). Beilstein Proceedings.

[22] Yang L., Li C., Song T., Zhan X. (2023). Growth hormone proteoformics atlas created to promote predictive, preventive, and personalized approach in overall management of pituitary neuroendocrine tumors. EPMA J..

[23] Zhan X., Li B., Zhan X., Schlüter H., Jungblut P.R., Coorssen J.R. (2019). Innovating the concept and practice of two-dimensional gel electrophoresis in the analysis of proteomes at the proteoform level. Proteomes.

[24] Zhan X., Li N., Zhan X., Qian S. (2018). Revival of 2DE-LC/MS in proteomics and its potential for large-scale study of human proteoforms. Med. One.

[25] Li N., Desiderio D.M., Zhan X. (2022). The use of mass spectrometry in a proteome-centered multiomics study of human pituitary adenomas. Mass. Spectrom. Rev..

[26] Zhan X., Su J., Yang L. (2023). Editorial: biomolecular modifications in endocrine-related cancers. Front. Endocrinol. (Lausanne).

[27] Evans W.E., Relling M.V. (1999). Pharmacogenomics: translating functional genomics into rational therapeutics. Science.

[28] Vesell E.S. (1989). Pharmacogenetic perspectives gained from twin and family studies. Pharmacol. Ther..

[29] Guengerich F.P., Hosea N.A., Parikh A., Bell-Parikh L.C., Johnson W.W., Gillam E.M. (1998). Twenty years of biochemistry of human P450s: purification, expression, mechanism, and relevance to drugs. Drug Metab. Dispos..

[30] Meyer U.A., Zanger U.M. (1997). Molecular mechanisms of genetic polymorphisms of drug metabolism. Annu. Rev. Pharmacol. Toxicol..

[31] Gonzalez F.J., Skoda R.C., Kimura S., Umeno M., Zanger U.M., Nebert D.W. (1988). Characterization of the common genetic defect in humans deficient in debrisoquine metabolism. Nature.

[32] Evans W.E., Johnson J.A. (2001). Pharmacogenomics: the inherited basis for interindividual differences in drug response. Annu. Rev. Genomics Hum. Genet..

[33] Weinshilboum R. (2003). Inheritance and drug response. N. Engl. J. Med..

[34] Evans W.E., McLeod H.L. (2003). Pharmacogenomics--drug disposition, drug targets, and side effects. N. Engl. J. Med..

[35] Hicks J.K., Dunnenberger H.M., Gumpper K.F., Haidar C.E., Hoffman J.M. (2016). Integrating pharmacogenomics into electronic health records with clinical decision support. Am. J. Health Syst. Pharm..

[36] Dunnenberger H.M., Biszewski M., Bell G.C., Sereika A., May H., Johnson S.G. (2016). Implementation of a multidisciplinary pharmacogenomics clinic in a community health system. Am. J. Health Syst. Pharm..

[37] Wake D.T., Ilbawi N., Dunnenberger H.M., Hulick P.J. (2019). Pharmacogenomics: prescribing precisely. Med. Clin. North Am..

[38] Roden D.M., McLeod H.L., Relling M.V., Williams M.S., Mensah G.A., Peterson J.F. (2019). Pharmacogenomics. Lancet.

[39] Evans W.E., Relling M.V. (2004). Moving towards individualized medicine with pharmacogenomics. Nature.

[40] Sadee W. (2011). Genomics and personalized medicine. Int. J. Pharm..

[41] Fielding J. (1980). History of penicillin. Lancet.

[42] Moberg C.L. (1991). Penicillin's forgotten man: norman Heatley. Science.

[43] Montinari M.R., Minelli S., De Caterina R. (2019). The first 3500 years of aspirin history from its roots - a concise summary. Vascul. Pharmacol..

[44] Desborough M.J.R., Keeling D.M. (2017). The aspirin story - from willow to wonder drug. Br. J. Haematol..

[45] Ittaman S.V., VanWormer J.J., Rezkalla S.H. (2014). The role of aspirin in the prevention of cardiovascular disease. Clin. Med. Res..

[46] Blumenthal K.G., Peter J.G., Trubiano J.A., Phillips E.J. (2019). Antibiotic allergy. Lancet.

[47] Lima L.M., Silva B.N.M.D., Barbosa G., Barreiro E.J. (2020). β-lactam antibiotics: an overview from a medicinal chemistry perspective. Eur. J. Med. Chem..

[48] Nicolaou K.C., Rigol S. (2018). A brief history of antibiotics and select advances in their synthesis. J. Antibiot. (Tokyo).

[49] Handin R.I. (2016). The history of antithrombotic therapy: the discovery of heparin, the vitamin K antagonists, and the utility of aspirin. Hematol. Oncol. Clin. North Am..

[50] Rachlis A.R. (1990). Zidovudine (Retrovir) update. CMAJ.

[51] Wong D.T., Perry K.W., Bymaster F.P. (2005). Case history: the discovery of fluoxetine hydrochloride (Prozac). Nat. Rev. Drug Discov..

[52] Argulian E., Bangalore S., Messerli F.H. (2019). Misconceptions and facts about beta-blockers. Am. J. Med..

[53] Cooper D.L., Braverman I.M., Sarris A.H., Durivage H.J., Saidman B.H., Davis C.A. (1993). Cyclosporine treatment of refractory T-cell lymphomas. Cancer.

[54] Sirtori C.R. (2014). The pharmacology of statins. Pharmacol. Res..

[55] Mathieu C., Gillard P., Benhalima K. (2017). Insulin analogues in type 1 diabetes mellitus: getting better all the time. Nat. Rev. Endocrinol..

[56] Heran B.S., Wong M.M., Heran I.K., Wright J.M. (2008). Blood pressure lowering efficacy of angiotensin converting enzyme (ACE) inhibitors for primary hypertension. Cochrane Database Syst. Rev..

[57] Diebel L.N., Liberati D.M., Hall-Zimmerman L. (2011). H2 blockers decrease gut mucus production and lead to barrier dysfunction *in vitro*. Surgery.

[58] Szuhany K.L., Simon N.M. (2022). Anxiety disorders: a review. JAMA.

[59] Grosser T., Ricciotti E., FitzGerald G.A. (2017). The Cardiovascular pharmacology of nonsteroidal anti-Inflammatory drugs. Trends Pharmacol. Sci..

[60] Schaffer L.V., Millikin R.J., Miller R.M., Anderson L.C., Fellers R.T., Ge Y. (2019). Identification and quantification of proteoforms by mass spectrometry. Proteomics.

[61] Zhan X., Yang H., Peng F., Li J., Mu Y., Long Y. (2018). How many proteins can be identified in a 2DE gel spot within an analysis of a complex human cancer tissue proteome?. Electrophoresis.

[62] Melani R.D., Gerbasi V.R., Anderson L.C., Sikora J.W., Toby T.K., Hutton J.E. (2022). The Blood Proteoform Atlas: a reference map of proteoforms in human hematopoietic cells. Science.

[63] Zhan X., Jungblut P.R. (2022). The comparison between 2DE-MS and bottom-up LC-MS demands high-end techniques for both technologies. Electrophoresis.

[64] Li B., Wang X., Yang C., Wen S., Li J., Li N. (2021). Human growth hormone proteoform pattern changes in pituitary adenomas: potential biomarkers for 3P medical approaches. EPMA J..

[65] Qian S., Yang Y., Li N., Cheng T., Wang X., Liu J. (2018). Prolactin variants in human pituitaries and pituitary adenomas identified with two-dimensional gel electrophoresis and mass spectrometry. Front. Endocrinol. (Lausanne).

[66] Zheng Y., Ley S.H., Hu F.B. (2018). Global aetiology and epidemiology of type 2 diabetes mellitus and its complications. Nat. Rev. Endocrinol..

[67] Frérot M., Lefebvre A., Aho S., Callier P., Astruc K., Aho Glélé L.S. (2018). What is epidemiology? Changing definitions of epidemiology 1978-2017. PLoS One.

[68] Relton C.L., Davey Smith G. (2010). Epigenetic epidemiology of common complex disease: prospects for prediction, prevention, and treatment. PLoS Med..

[69] Ferlay J., Colombet M., Soerjomataram I., Dyba T., Randi G., Bettio M. (2018). Cancer incidence and mortality patterns in Europe: estimates for 40 countries and 25 major cancers in 2018. Eur. J. Cancer.

[70] Wu F., Wang L., Zhou C. (2021). Lung cancer in China: current and prospect. Curr. Opin. Oncol..

[71] Fedorov, Lineva V.I., Tarasova I.A., Gorshkov M.V. (2022). Mass spectrometry-based chemical proteomics for drug target discoveries. Biochemistry (Mosc.).

[72] Huang F., Zhang B., Zhou S., Zhao X., Bian C., Wei Y. (2012). Chemical proteomics: terra incognita for novel drug target profiling. Chin. J. Cancer.

[73] Gianazza E., Brioschi M., Baetta R., Mallia A., Banfi C., Tremoli E. (2020). Platelets in healthy and disease states: from biomarkers discovery to drug targets identification by proteomics. Int. J. Mol. Sci..

[74] Zhang W., Wang T. (2023). Understanding protein functions in the biological context. Protein Pept. Lett..

[75] Aslam B., Basit M., Nisar M.A., Khurshid M., Rasool M.H. (2017). Proteomics: technologies and their applications. J. Chromatogr. Sci..

[76] Monti C., Zilocchi M., Colugnat I., Alberio T. (2019). Proteomics turns functional. J. Proteomics.

[77] Mendes M.L., Dittmar G. (2022). Targeted proteomics on its way to discovery. Proteomics.

[78] Aebersold R., Mann M. (2016). Mass-spectrometric exploration of proteome structure and function. Nature.

[79] Gallien S., Duriez E., Demeure K., Domon B. (2013). Selectivity of LC-MS/MS analysis: implication for proteomics experiments. J. Proteomics.

[80] Gemmete J.J., Mukherji S.K. (2011). Trastuzumab (herceptin). AJNR Am. J. Neuroradiol..

[81] Carpenter G., King L., Cohen S. (1978). Epidermal growth factor stimulates phosphorylation in membrane preparations *in vitro*. Nature.

[82] Schechter A.L., Stern D.F., Vaidyanathan L., Decker S.J., Drebin J.A., Greene M.I. (1984). The neu oncogene: an erb-B-related gene encoding a 185,000-Mr tumour antigen. Nature.

[83] Kyaw A. (1978). Proteinase and protease. Lancet.

[84] Reina J., Iglesias C. (2022). Nirmatrelvir plus ritonavir (Paxlovid) a potent SARS-CoV-2 3CLpro protease inhibitor combination. Rev. Esp. Quimioter..

[85] Dai W., Zhang B., Jiang X.M., Su H., Li J., Zhao Y. (2020). Structure-based design of antiviral drug candidates targeting the SARS-CoV-2 main protease. Science.

[86] Chia C.S.B., Xu W., Shuyi Ng P. (2022). A patent review on SARS coronavirus main protease (3CLpro) inhibitors. ChemMedChem.

[87] Estébanez M., Arribas J.R. (2012). Protease inhibitor monotherapy: what is its role?. Curr. HIV/AIDS Rep..

[88] Bierman W.F., van Agtmael M.A., Nijhuis M., Danner S.A., Boucher C.A. (2009). HIV monotherapy with ritonavir-boosted protease inhibitors: a systematic review. AIDS.

[89] Pérez-Valero I., Arribas J.R. (2011). Protease inhibitor monotherapy. Curr. Opin. Infect. Dis..

[90] Xu J., Zhang J., Wang L., Zhou J., Huang H., Wu J. (2006). Solution structure of Urm1 and its implications for the origin of protein modifiers. Proc. Natl. Acad. Sci. U. S. A..

[91] Singh R.K., Lee J.K., Selvaraj C., Singh R., Li J., Kim S.Y. (2018). Protein engineering approaches in the post-genomic era. Curr. Protein Pept. Sci..

[92] Hershko A., Ciechanover A. (1998). The ubiquitin system. Annu. Rev. Biochem..

[93] Chen Z.J., Parent L., Maniatis T. (1996). Site-specific phosphorylation of IkappaBalpha by a novel ubiquitination-dependent protein kinase activity. Cell.

[94] Schwartz D.C., Hochstrasser M. (2003). A superfamily of protein tags: ubiquitin, SUMO and related modifiers. Trends Biochem. Sci..

[95] Salmaninejad A., Valilou S.F., Shabgah A.G., Aslani S., Alimardani M., Pasdar A. (2019). PD-1/PD-L1 pathway: basic biology and role in cancer immunotherapy. J. Cell. Physiol..

[96] Ljunggren H.G., Jonsson R., Höglund P. (2018). Seminal immunologic discoveries with direct clinical implications: the 2018 Nobel Prize in Physiology or Medicine honours discoveries in cancer immunotherapy. Scand. J. Immunol..

[97] Parkin J., Cohen B. (2001). An overview of the immune system. Lancet.

[98] Rowshanravan B., Halliday N., Sansom D.M. (2018). CTLA-4: a moving target in immunotherapy. Blood.

[99] Han Y., Liu D., Li L. (2020). PD-1/PD-L1 pathway: current researches in cancer. Am. J. Cancer Res..

[100] Manansala M., Baughman R., Novak R., Judson M., Sweiss N. (2021). Management of immunosuppressants in the era of coronavirus disease-2019. Curr. Opin. Pulm. Med..

[101] Di Maira T., Little E.C., Berenguer M. (2020). Immunosuppression in liver transplant. Best Pract. Res. Clin. Gastroenterol..

[102] Houston S. (2022). Immunosuppression. Nat. Immunol..

[103] Riley R.S., June C.H., Langer R., Mitchell M.J. (2019). Delivery technologies for cancer immunotherapy. Nat. Rev. Drug Discov..

[104] Zhang Y., Zhang Z. (2020). The history and advances in cancer immunotherapy: understanding the characteristics of tumor-infiltrating immune cells and their therapeutic implications. Cell. Mol. Immunol..

[105] Carbonara K., Andonovski M., Coorssen J.R. (2021). Proteomes are of proteoforms: embracing the complexity. Proteomes.

[106] Jungblut P.R., Thiede B., Schlüter H. (2016). Towards deciphering proteomes *via* the proteoform, protein speciation, moonlighting and protein code concepts. J. Proteomics.

[107] Gorr T.A., Vogel J. (2015). Western blotting revisited: critical perusal of underappreciated technical issues. Proteomics Clin. Appl..

[108] Anjo S.I., Santa C., Manadas B. (2017). SWATH-MS as a tool for biomarker discovery: from basic research to clinical applications. Proteomics.

[109] Issaq H.J., Veenstra T.D. (2007). The role of electrophoresis in disease biomarker discovery. Electrophoresis.

[110] Xu H., Wang Y., Lin S., Deng W., Peng D., Cui Q. (2018). PTMD: a Database of human disease-associated post-translational modifications. Genomics Proteomics Bioinformatics.

[111] Wen S., Li C., Zhan X. (2022). Muti-omics integration analysis revealed molecular network alterations in human nonfunctional pituitary neuroendocrine tumors in the framework of 3P medicine. EPMA J..

[112] Golubnitschaja O., Potuznik P., Polivka J., Pesta M., Kaverina O., Pieper C.C. (2022). Ischemic stroke of unclear aetiology: a case-by-case analysis and call for a multi-professional predictive, preventive and personalised approach. EPMA J..

[113] Koklesova L., Mazurakova A., Samec M., Kudela E., Biringer K., Kubatka P. (2022). Mitochondrial health quality control: measurements and interpretation in the framework of predictive, preventive, and personalized medicine. EPMA J..

[114] Evsevieva M., Sergeeva O., Mazurakova A., Koklesova L., Prokhorenko-Kolomoytseva I., Shchetinin E. (2022). Pre-pregnancy check-up of maternal vascular status and associated phenotype is crucial for the health of mother and offspring. EPMA J..

[115] Kanaka V., Proikakis S., Drakakis P., Loutradis D., Tsangaris G.T. (2022). Implementing a preimplantation proteomic approach to advance assisted reproduction technologies in the framework of predictive, preventive, and personalized medicine. EPMA J..

[116] Xu J., Zhou H., Cheng Y., Xiang G. (2022). Identifying potential signatures for atherosclerosis in the context of predictive, preventive, and personalized medicine using integrative bioinformatics approaches and machine-learning strategies. EPMA J..

[117] Kurysheva N.I., Rodionova O.Y., Pomerantsev A.L., Sharova G.A., Golubnitschaja O. (2023). Machine learning-couched treatment algorithms tailored to individualized profile of patients with primary anterior chamber angle closure predisposed to the glaucomatous optic neuropathy. EPMA J..

[118] Schwinn D.A., Afshari N.A. (2006). alpha(1)-Adrenergic receptor antagonists and the iris: new mechanistic insights into floppy iris syndrome. Surv. Ophthalmol..

[119] Smith S.A., Smith S.E. (1980). Subsensitivity to cholinoceptor stimulation of the human iris sphincter *in situ* following acute and chronic administration of cholinomimetic miotic drugs. Br. J. Pharmacol..

[120] Alm A., Nilsson S.F. (2009). Uveoscleral outflow--a review. Exp. Eye Res..

[121] McDevitt D.G. (1989). *In vivo* studies on the function of cardiac beta-adrenoceptors in man. Eur. Heart J..

[122] Karagueuzian H.S. (2011). How does cholinergic activation slow down sinus node automaticity? “diastolic voltage oscillations” vs. “calcium clock” mechanisms. J. Cardiovasc. Electrophysiol..

[123] Frishman W.H. (1988). Circulatory and metabolic aspects of beta-adrenoceptor blockade. Am. Heart J..

[124] Higgins C.B., Vatner S.F., Braunwald E. (1973). Parasympathetic control of the heart. Pharmacol. Rev..

[125] Stöckigt F., Brixius K., Lickfett L., Andrié R., Linhart M., Nickenig G. (2012). Total beta-adrenoceptor knockout slows conduction and reduces inducible arrhythmias in the mouse heart. PLoS One.

[126] Halder N., Lal G. (2021 15). Cholinergic system and its therapeutic importance in inflammation and autoimmunity. Front. Immunol..

[127] Wallukat G. (2002). The beta-adrenergic receptors. Herz.

[128] Kilter H., Lenz O., La Rosée K., Flesch M., Schwinger R.H., Mädge M. (1995). Evidence against a role of nitric oxide in the indirect negative inotropic-effect of M-cholinoceptor stimulation in human ventricular myocardium. Naunyn Schmiedebergs Arch. Pharmacol..

[129] Brown M.J. (1989). Investigation of alpha 2-adrenoceptors in humans. Am. J. Med..

[130] McVary K.T., McKenna K.E., Lee C. (1998). Prostate innervation. Prostate Suppl..

[131] Harrison D.G., Sellke F.W., Quillen J.E. (1990). Neurohumoral regulation of coronary collateral vasomotor tone. Basic Res. Cardiol..

[132] Shiuchi T., Toda C., Okamoto S., Coutinho E.A., Saito K., Miura S. (2017). Induction of glucose uptake in skeletal muscle by central leptin is mediated by muscle β2-adrenergic receptor but not by AMPK. Sci. Rep..

[133] Rodrigues A.C.Z., Messi M.L., Wang Z.M., Abba M.C., Pereyra A., Birbrair A. (2019). The sympathetic nervous system regulates skeletal muscle motor innervation and acetylcholine receptor stability. Acta Physiol. (Oxf).

[134] Rangaraj N., Kalant H., Beaugé F. (1985). Alpha 1-adrenergic receptor involvement in norepinephrine-ethanol inhibition of rat brain Na+ -K+ ATPase and in ethanol tolerance. Can J. Physiol. Pharmacol..

[135] Kamiar A., Yousefi K., Dunkley J.C., Webster K.A., Shehadeh L.A. (2021). β2-Adrenergic receptor agonism as a therapeutic strategy for kidney disease. Am. J. Physiol. Regul. Integr. Comp. Physiol..

[136] Nakamura A., Imaizumi A., Yanagawa Y. (2004). [Beta 2-adrenoceptor function in the kidney]. Nihon Yakurigaku Zasshi.

[137] Whitehead A.K., Erwin A.P., Yue X. (2021). Nicotine and vascular dysfunction. Acta Physiol. (Oxf).

[138] Lulich K.M., Goldie R.G., Paterson J.W. (1988). Beta-adrenoceptor function in asthmatic bronchial smooth muscle. Gen. Pharmacol..

[139] Hvizdos K.M., Goa K.L. (2002). Tiotropium bromide. Drugs.

[140] Ahlman H., Nilsson O., Wängberg B., Dahlström A. (1996). Neuroendocrine insights from the laboratory to the clinic. Am. J. Surg..

[141] Niederberger M.D., Hirsbrunner G., Steiner A., Brechbühl M., Meylan M. (2010). *In vitro* effects of bethanechol on abomasal and duodenal smooth muscle preparations from dairy cows with left displacement of the abomasum and from healthy dairy cows. Vet. J..

[142] Gomes L.T.D.C., de Sena M.O., Dantas P.B., Barbosa A.I.D.S., Holanda V.A.D., Oliveira J.I.N. (2023 1). Smooth muscle contraction of the fundus of stomach, duodenum and bladder from mice exposed to a stress-based model of depression. Physiol. Behav..

[143] Lundgren O. (1983). Vagal control of the motor functions of the lower esophageal sphincter and the stomach. J. Auton. Nerv. Syst..

[144] Lipworth B.J. (1996). Clinical pharmacology of beta 3-adrenoceptors. Br. J. Clin. Pharmacol..

[145] Lee M.C., Yang Y.C., Chen Y.C., Huang S.C. (2013). Muscarinic receptor M3 mediates human gallbladder contraction through voltage-gated Ca2+ channels and Rho kinase. Scand. J. Gastroenterol..

[146] Michel M.C., Vrydag W. (2006). Alpha1-, alpha2- and beta-adrenoceptors in the urinary bladder, urethra and prostate. Br. J. Pharmacol..

[147] Yamanishi T., Chapple C.R., Chess-Williams R. (2001). Which muscarinic receptor is important in the bladder?. World J. Urol..

[148] Jolliet P., Bourin M. (1998). Urosélectivité de l'antagonisme alpha-1 dans le traitement de l'hypertrophie bénigne de la prostate: du concept pharmacologique à l'approche clinique [Uroselectivity of alpha-1 antagonism in the treatment of benign prostatic hypertrophy: on the pharmacologic concept of the clinical approach. Therapie.

[149] Williams L.T., Mullikin D., Lefkowitz R.J. (1976). Identification of alpha-adrenergic receptors in uterine smooth muscle membranes by [3H]dihydroergocryptine binding. J. Biol. Chem..

[150] Kitazawa T., Hirama R., Masunaga K., Nakamura T., Asakawa K., Cao J. (2008). Muscarinic receptor subtypes involved in carbachol-induced contraction of mouse uterine smooth muscle. Naunyn Schmiedebergs Arch. Pharmacol..

[151] Bellavía S., Gallará R. (2000). Effect of photic stimuli on rat salivary glands. Role of sympathetic nervous system. Acta Odontol. Latinoam..

[152] Proctor G.B., Carpenter G.H. (2007). Regulation of salivary gland function by autonomic nerves. Auton. Neurosci..

[153] Bone R.C., Hiller C. (1978). Modern treatment of bronchial asthma. JACEP.

[154] Goldie R.G. (1990). Receptors in asthmatic airways. Am. Rev. Respir. Dis..

[155] Goldie R.G., Grayson P.S., Knott P.G., Self G.J., Henry P.J. (1994). Predominance of endothelinA (ETA) receptors in ovine airway smooth muscle and their mediation of ET-1-induced contraction. Br. J. Pharmacol..

[156] Hu Y., Converse C., Lyons M.C., Hsu W.H. (2018). Neural control of sweat secretion: a review. Br. J. Dermatol..

[157] Longmore J., Bradshaw C.M., Szabadi E. (1985). Effects of locally and systemically administered cholinoceptor antagonists on the secretory response of human eccrine sweat glands to carbachol. Br. J. Clin. Pharmacol..

[158] Shepherd J.T. (1985). Circulatory response to beta-adrenergic blockade at rest and during exercise. Am. J. Cardiol..

[159] Mussina K., Toktarkhanova D., Filchakova O. (2021). Nicotinic acetylcholine receptors of PC12 cells. Cell. Mol. Neurobiol..

[160] Exton J.H. (1987). Mechanisms of hormonal regulation of hepatic glucose metabolism. Diabetes Metab. Rev..

[161] (2017). LiverTox: Clinical and Research Information on Drug-Induced Liver Injury [Internet].

[162] Yang L.K., Tao Y.X. (2019). Physiology and pathophysiology of the β3-adrenergic receptor. Prog. Mol. Biol. Transl. Sci..

[163] Lynch G.S., Ryall J.G. (2008). Role of beta-adrenoceptor signaling in skeletal muscle: implications for muscle wasting and disease. Physiol. Rev..

[164] Soendenbroe C., Heisterberg M.F., Schjerling P., Kjaer M., Andersen J.L., Mackey A.L. (2022). Human skeletal muscle acetylcholine receptor gene expression in elderly males performing heavy resistance exercise. Am. J. Physiol. Cell Physiol..

